# Biofilm formation as a novel phenotypic feature of adherent-invasive *Escherichia coli *(AIEC)

**DOI:** 10.1186/1471-2180-9-202

**Published:** 2009-09-21

**Authors:** Margarita Martinez-Medina, Plínio Naves, Jorge Blanco, Xavier Aldeguer, Jesus E Blanco, Miguel Blanco, Carmen Ponte, Francisco Soriano, Arlette Darfeuille-Michaud, L Jesus Garcia-Gil

**Affiliations:** 1 Laboratory of Molecular Microbiology, Biology Department, University of Girona, Spain; 2 Department of Medical Microbiology and Antimicrobial Chemotherapy, Fundación Jiménez Díaz-Capio, Madrid, Spain; 3 E. coli Reference Laboratory, Faculty of Veterinary, University of Santiago de Compostela, Lugo, Spain; 4 Gastroenterology Department, Dr. Josep Trueta Hospital, Girona, Spain; 5 Laboratory of Intestinal Bacterial Pathogenesis, USC-INRA 2018, JE 2526 University of Auvergne, Clermont-Ferrand, France

## Abstract

**Background:**

Crohn's disease (CD) is a high morbidity chronic inflammatory disorder of unknown aetiology. Adherent-invasive *Escherichia coli *(AIEC) has been recently implicated in the origin and perpetuation of CD. Because bacterial biofilms in the gut mucosa are suspected to play a role in CD and biofilm formation is a feature of certain pathogenic *E. coli *strains, we compared the biofilm formation capacity of 27 AIEC and 38 non-AIEC strains isolated from the intestinal mucosa. Biofilm formation capacity was then contrasted with the AIEC phenotype, the serotype, the phylotype, and the presence of virulence genes.

**Results:**

Specific biofilm formation (SBF) indices were higher amongst AIEC than non-AIEC strains (P = 0.012). In addition, 65.4% of moderate to strong biofilms producers were AIEC, whereas 74.4% of weak biofilm producers were non-AIEC (P = 0.002). These data indicate that AIEC strains were more efficient biofilm producers than non-AIEC strains. Moreover, adhesion (P = 0.009) and invasion (P = 0.003) indices correlated positively with higher SBF indices. Additionally, motility (100%, P < 0.001), H1 type flagellin (53.8%, P < 0.001), serogroups O83 (19.2%, P = 0.008) and O22 (26.9%, P = 0.001), the presence of virulence genes such as *sfa/focDE *(38.5%, P = 0.003) and *ibeA *(26.9%, P = 0.017), and B2 phylotype (80.8%, P < 0.001) were frequent characteristics amongst biofilm producers.

**Conclusion:**

The principal contribution of the present work is the finding that biofilm formation capacity is a novel, complementary pathogenic feature of the recently described AIEC pathovar. Characterization of AIEC specific genetic determinants, and the regulatory pathways, involved in biofilm formation will likely bring new insights into AIEC pathogenesis.

## Background

Crohn's disease (CD) is a chronic-relapsing inflammatory bowel disease (IBD) that can affect the entire gastrointestinal tract. The incidence rate varies from 1 to 20 cases per 10^5 ^people per year and is still rising in some countries [1]. Although the aetiology of CD remains elusive to date, it is widely accepted that several factors are involved in the onset or perpetuation of the disease. These factors include genetic and immunologic features that confer host susceptibility, and external or environmental factors such as microorganisms and lifestyle [2,3]. Environmental factors play an important role because there is a low concordance between identical twins, both for CD and ulcerative colitis (UC) [4]. The involvement of microbes in the onset or perpetuation of inflammation has been extensively studied [5-10]. To date, some pathogens have been proposed as causative agents. In particular, adherent-invasive *E. coli *(AIEC) is increasing in relevance because it has been reported to be more prevalent in CD patients than in controls in several countries (France [11], United Kingdom [12], USA [13,14], and Spain [15]). AIEC strains have the ability to adhere to and to invade intestinal epithelial cells *in vitro *as well as to survive and replicate within macrophages without inducing host-cell death and promoting tumour necrosis factor (TNF) α release. No unique genetic sequences have been described for AIEC, nor have specific genes of diarrhoeagenic pathovars been detected yet for AIEC, but they do carry many virulence-associated genes characteristic of extraintestinal pathogenic *E. coli *(ExPEC) [13,15,16]. For that reason, AIEC pathovar has been speculated to be closely related to ExPEC pathovar.

In a previous work, we observed that some CD patients showed a high diversity of AIEC subtypes associated to their intestinal mucosa [15]. In a given patient, we could detect up to 8 different clones as assessed by pulsed field gel electrophoresis. On the other hand, AIEC abundance, richness and diversity were lower in non-IBD controls. We hypothesized that the higher diversification of clones could be explained by a long-term colonization of AIEC in CD. Biofilm formation can be a way to persistently colonize the intestinal mucosa [17], as has been reported for commensal microbiota in healthy subjects [18]. Moreover, for certain organisms such as the species belonging to the uropathogenic *E. coli *pathovar (UPEC) - which form intracellular biofilms [19] - and to the enteroaggregative pathogenic *E. coli *pathovar (EAEC) - which form thick biofilms that adhere to the apical side of enterocytes [20]-, active biofilm formation is feature of their pathogenesis. For that reason, the primarily aim of this work was to determine the biofilm formation capacity of AIEC strains and non-AIEC strains, both isolated from the intestinal mucosa.

We herein report a new phenotypic feature of the recently described AIEC pathovar which is the ability to form biofilms *in vitro*. In addition, we illustrate those seropathotypes and phylotypes more frequently found amongst biofilm producers.

## Results

### AIEC strains are stronger biofilm producers than non-AIEC strains isolated from the intestinal mucosa

The present study involved a collection of 65 *E. coli *strains, 27 of which (41.5%) were classified as AIEC by their ability to adhere to and invade intestinal epithelial cells, and to survive and replicate within macrophages, as previously described [11] (Table 1).

**Table 1:** Phenotypic and genotypic characteristics of the bacterial strains used in this study.

Id Strain	Origin	Serotype	Phylo.	Virulence gene carriage
AIEC19	C	ONT: H-	A	*iucD, fimH, fimAv*_*MT*78_

AIEC07	C	O22: H7	B1	*papC, iucD, fimH*

AIEC04	C	O6: HNT	B2	*papC, sfa/focDE, iucD, hlyA, cnf1, fimH, fimAv*_*MT*78_

AIEC10	C	O159: H34	A	*fimH*

AIEC06	C	O6: H5	B2	*papC, sfa/focDE, hlyA, cnf1, fimH, cdtB*

AIEC08	C	O25:H4	B2	*papC, iucD, ibeA, fimH, cdtB*

AIEC25	CD	O6: H31	B2	*papC, hlyA, cnf1, fimH*

AIEC21	CD	O6: H1	B2	*papC, iucD, hlyA, cnf1, fimH*

AIEC12	CD	O26: H-	B2	*papC, afa/draBC, iucD, fimH*

AIEC20	CD	O11: H18	D	*papC, afa/draBC, iucD, fimH*

AIEC17	CD	ONT: HNT	D	*neuC, ibeA, fimH*

AIEC05	CD	O1: H-	B2	*papC, neuC, fimH*

AIEC02	CD	O8: H21	B2	*papC, neuC, fimH*

AIEC01	CD	O6: H1	B2	*sfa/focDE, iucD, fimH*

AIEC09	CD	ONT: H-	B2	*papC, sfa/focDE, iucD, hlyA, cnf1, fimH, fimAv*_*MT*78_

AIEC24	CD	ONT: H-	A	*iucD, fimH, fimAv*_*MT*78_

AIEC23	CD	O5: HNT	A	*fimH*

AIEC11	CD	O22: H1	B2	*afa/draBC, iucD, fimH*

AIEC15-1	CD	O22: H1	B2	*fimH*

AIEC14-1	CD	O22: H1	B2	*fimH*

AIEC16-2	CD	O22: H1	B2	*fimH*

LF82	CD	O83: H1	B2	*ibeA, fimH, fimAv*_*MT*78_

AIEC13	UC	O25: H4	B2	*papC, iucD, hlyA, cnf1, ibeA, fimH, cdtB*

PP16	Sepsis	O83: H1	B2	*ibeA, fimH, fimAv*_*MT*78_

FV7563	UTI	O25: H4	B2	*afa/draBC, iucD, fimH*

OL96A	UTI	O6: H1	B2	*papC, sfa/focDE, iucD, hlyA, cnf1, fimH*

PP215	Sepsis	O6: H1	B2	*papC, sfa/focDE, iucD, hlyA, cnf1, fimH*

ECG-046	C	ONT: H29	B1	*iucD*, pCDV432

ECG-060	C	O102: H6	D	*papC, iucD, fimH*

ECG-037	C	O1: H-	D	*papC, neuC, iucD, fimH, fimAv*_*MT*78_

ECG-016	C	O55: H-	A	*neuC, iucD, fimH*

ECG-017	C	O22: H18	B2	*sfa/focDE, fimH*

ECG-022	C	O15: H-	A	*afa/draBC, neuC, iucD, fimH*

ECG-043	C	O83: H1	B2	*ibeA, fimH, fimAv*_*MT*78_

ECG-041	C	O2: H-	B2	*papC, sfa/focDE, iucD, hlyA, cnf1, fimH, cdtB*

ECG-012	C	O18: H1	B2	*papC, sfa/focDE, iucD, hlyA, cnf1, fimH*

ECG-025	C	O6: H31	B2	*papC, hlyA, cnf1, fimH*

ECG-049	C	O15: H16	B2	*papC, iucD, fimH*

ECG-031	C	O161: H4	D	*iucD, fimH*

ECG-023	C	ONT: H-	At	*iucD, fimH, fimAv*_*MT*78_

ECG-054	C	O14: H-	D	*papC, iucD, fimH*

ECG-008	C	O14: H7	B2	*papC, iucD, ibeA, fimH, fimAv*_*MT*78_

ECG-004	C	O22: H7	B1	*fimH*

ECG-013	C	O18: H1	B2	*papC, sfa/focDE, iucD, hlyA, cnf1, fimH*

ECG-055	C	O17: H18	D	*papC, iucD, fimH*

ECG-024	C	O174: H-	B1	*fimH*

ECG-064	CD	O166: H21	B1	*fimH*

ECG-042	CD	O7: H6	B2	*fimH, fimAv*_*MT*78_

ECG-001	CD	O6: H31	B2	*papC, hlyA, cnf1, fimH*

ECG-005	CD	O4: H5	B2	*papC, sfa/focDE, hlyA, cnf1, fimH*

ECG-065	CD	O141: H-	A	*fimH, fimAv*_*MT*78_

ECG-047	CD	O119: H21	D	*iucD, fimH*

ECG-019	CD	ONT: H-	A	

ECG-018	CD	ONT: H-	A	*iucD*

ECG-002	CD	O175: H-	B1	*fimH*

ECG-034	CD	O127: H28	D	*iucD, fimH*

ECG-021	CD	O54: H21	B1	*fimH*

ECG-063	CD	ONT: H21	B1	*fimH*

ECG-056	CD	ONT: H18	D	*papC, afa/draBC, iucD, fimH*

ECG-057	CD	O11: H18	D	*papC, afa/draBC, iucD, fimH*

ECG-053	CD	O11: H-	D	*papC, afa/draBC, iucD, fimH*

ECG-059	CD	ONT: H18	D	*papC, iucD, fimH*

ECG-026	CD	O1: H-	B2	*papC, neuC, ibeA, fimH, fimAv*_*MT*78_

ECG-015	CD	O2: H6	B2	*papC, sfa/focDE, neuC, hlyA, cnf1, fimH*

ECG-009	CD	O83: H1	B2	*sfa/focDE, neuC, iucD, ibeA, fimH, fimAv*_*MT*78_,*cdtB*

Abbreviators: CD: Crohn's disease; UC: ulcerative colitis; C: non-IBD control, UTI: urinary tract infection; ONT: serogroup non-typeable; HNT: flagellar antigen non-typeable; H-: nonmotile strain; Phylo.: Phylogroup; At: Atypical.

**Figure 1:**
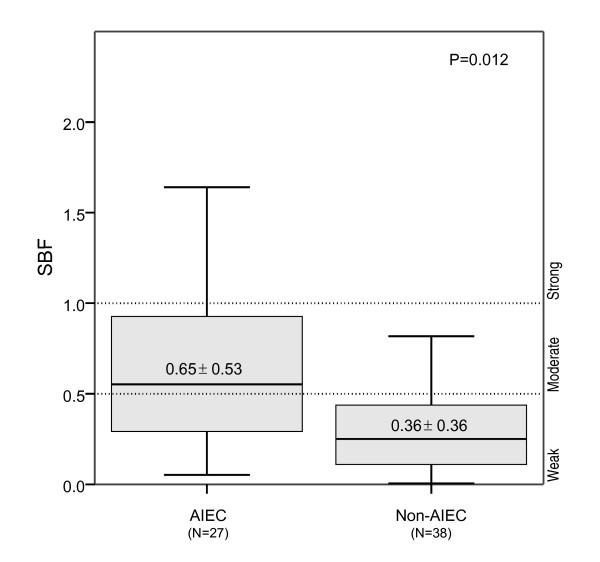
**Mean specific biofilm formation (SBF) index of AIEC and mucosa-associated non-AIEC strains**. The mean SBF index was higher for AIEC than for non-AIEC strains, as corroborated by one-way ANOVA (P = 0.012).

Within the category of weak biofilm producers, 74.4% of strains were non-AIEC, whereas 65.4% of moderate to strong biofilm producers were AIEC (P = 0.002). Amongst these AIEC strains, 22.2% were strong biofilm producers, and 40.7% were moderate biofilm producers (Table 2). Similar results were obtained when SBF index values were compared. As shown in Figure 1, the mean SBF index was higher in AIEC strains than in non-AIEC (SBF_AIEC _= 0.65 ± 0.53; SBF_NON-AIEC _= 0.36 ± 0.36; P = 0.012).

**Table 2:** AIEC phenotype and category of biofilm formation ability of the strains.

Id Strain	AIEC	I_ADH (mean ± SD)		I_INV (mean ± SD)		I_REPL (mean ± SD)		SBF (mean ± SD)		BFC
AIEC19	+	2.40	± 0.65	0.111	± 0.016	1568.1	± 1726	0.053	± 0.019	W

AIEC07	+	20.00	± 13.50	0.565	± 0.392	1692.6	± 296.8	2.391	± 0.317	S

AIEC04	+	21.50	± 9.00	0.320	± 0.016	584.7	± 418.5	0.772	± 0.132	M

AIEC10	+	6.00	± 0.98	0.226	± 0.192	1413.7	± 51.4	0.610	± 0.175	M

AIEC06	+	10.25	± 3.25	0.177	± 0.019	1717.7	± 307.9	1.212	± 0.233	S

AIEC08	+	1.13	± 0.18	0.172	± 0.066	104.8	± 49.7	0.397	± 0.133	W

AIEC25	+	2.75	± 1.33	0.482	± 0.129	775.9	± 128.3	0.437	± 0.129	W

AIEC21	+	17.00	± 7.75	0.109	± 0.013	1297.1	± 625.2	0.558	± 0.205	M

AIEC12	+	22.25	± 4.00	0.142	± 0.017	193.7	± 55.9	0.125	± 0.052	W

AIEC20	+	14.25	± 6.25	0.125	± 0.098	343.9	± 244.6	0.284	± 0.116	W

AIEC17	+	21.75	± 17.50	0.266	± 0.055	1053.0	± 75.0	0.840	± 0.286	M

AIEC05	+	9.50	± 2.25	0.202	± 0.042	704.9	± 714.0	0.181	± 0.072	W

AIEC02	+	0.85	± 1.03	0.802	± 0.035	2187.8	± 4.8	0.106	± 0.035	W

AIEC01	+	16.00	± 9.25	0.284	± 0.106	1566.7	± 1060	0.700	± 0.177	M

AIEC09	+	5.25	± 4.00	0.216	± 0.010	2562.3	± 240.6	0.068	± 0.035	W

AIEC24	+	1.98	± 1.40	0.309	± 0.138	1625.6	± 115.6	0.076	± 0.044	W

AIEC23	+	9.75	± 0.70	0.568	± 0.148	2362.1	± 250.2	0.300	± 0.093	W

AIEC11	+	0.83	± 0.19	2.125	± 1.164	739.4	± 477.4	0.537	± 0.129	M

AIEC15-1	+	25.00	± 15.75	2.261	± 1.349	776.9	± 304.8	1.090	± 0.407	S

AIEC14-1	+	4.25	± 3.50	0.508	± 0.081	847.9	± 512.8	0.654	± 0.153	M

AIEC16-2	+	10.00	± 1.425	0.305	± 0.159	659.7	± 437.0	0.502	± 0.134	M

LF82	+	25.00	± 5.25	2.261	± 0.011	776.9	± 252.4	1.641	± 0.326	S

AIEC13	+	1.20	± 4.25	0.104	± 0.000	1045.9	± 181.6	0.772	± 0.211	M

PP16	+	8.00	± 0.98	1.400	± 0.081	225.9	± 541.2	1.012	± 0.268	S

FV7563	+	6.75	± 6.00	0.129	± 0.072	470.0	± 264.0	0.518	± 0.226	M

OL96A	+	5.25	± 5.00	0.388	± 0.159	457.5	± 259.3	1.208	± 0.202	S

PP215	+	0.83	± 0.60	0.453	± 0.350	1425.4	± 229.4	0.546	± 0.139	M

ECG-046	-	-		< 0.1		-		0.004	± 0.010	W

ECG-060	-	-		< 0.1		-		0.127	± 0.041	W

ECG-037	-	-		< 0.1		-		0.042	± 0.024	W

ECG-016	-	-		< 0.1		-		0.134	± 0.085	W

ECG-017	-	-		< 0.1		-		1.074	± 0.286	S

ECG-022	-	-		< 0.1		-		0.143	± 0.090	W

ECG-043	-	-		< 0.1		-		1.187	± 0.511	S

ECG-041	-	-		< 0.1		-		0.301	± 0.123	W

ECG-012	-	-		< 0.1		-		0.741	± 0.259	M

ECG-025	-	-		< 0.1		-		0.154	± 0.043	W

ECG-049	-	-		< 0.1		-		0.384	± 0.160	W

ECG-031	-	-		< 0.1		-		0.067	± 0.024	W

ECG-023	-	0.90	± 0.65	0.052	± 0.003	-		0.038	± 0.020	W

ECG-054	-	-		< 0.1		-		0.209	± 0.128	W

ECG-008	-	-		< 0.1		-		0.817	± 0.288	M

ECG-004	-	-		< 0.1		-		1.113	± 0.234	S

ECG-013	-	-		< 0.1		-		0.516	± 0.332	M

ECG-055	-	-		< 0.1		-		0.108	± 0.033	W

ECG-024	-	-		< 0.1		-		0.037	± 0.016	W

ECG-064	-	-		< 0.1		-		0.553	± 0.171	M

ECG-042	-	-		< 0.1		-		0.348	± 0.147	W

ECG-001	-	-		< 0.1		-		0.299	± 0.106	W

ECG-005	-	-		< 0.1		-		0.404	± 0.103	W

ECG-065	-	-		0.061	± 0.070	-		0.026	± 0.022	W

ECG-047	-	1.93	± 1.95	0.259	± 0.084	-		0.007	± 0.016	W

ECG-019	-	-		< 0.1		-		0.439	± 0.057	W

ECG-018	-	-		< 0.1		-		0.058	± 0.042	W

ECG-002	-	-		< 0.1		-		0.039	± 0.023	W

ECG-034	-	-		< 0.1		-		0.293	± 0.101	W

ECG-021	-	6.00	± 4.00	0.033	± 0.011	-		0.311	± 0.117	W

ECG-063	-	-		< 0.1		-		0.195	± 0.064	W

ECG-056	-	-		< 0.1		-		0.124	± 0.047	W

ECG-057	-	11.75	± 7.25	0.013	± 0.011	-		0.241	± 0.094	W

ECG-053	-	-		< 0.1		-		0.262	± 0.083	W

ECG-059	-	-		< 0.1		-		0.200	± 0.137	W

ECG-026	-	-		< 0.1		-		0.418	± 0.189	W

ECG-015	-	5.25	± 2.75	0.038	± 0.004	-		1.035	± 0.219	S

ECG-009	-	-		< 0.1		-		1.346	± 0.205	S

Adhesion, invasion, intra-macrophage replication, and biofilm formation indices are specified. Abbreviators: AIEC: AIEC phenotype (+: strains that adhere to and invade Intestine-407 cells and that were able to survive and/or replicate within J774 macrophages *in vitro*); I_ADH: adhesion index; I_INV: invasion index; I_REPL: replication index; SBF: specific biofilm formation index; BFC: Biofilm formation category; W: weak biofilm producer; M: moderate biofilm producer; and S: strong biofilm producer.

**Figure 2:**
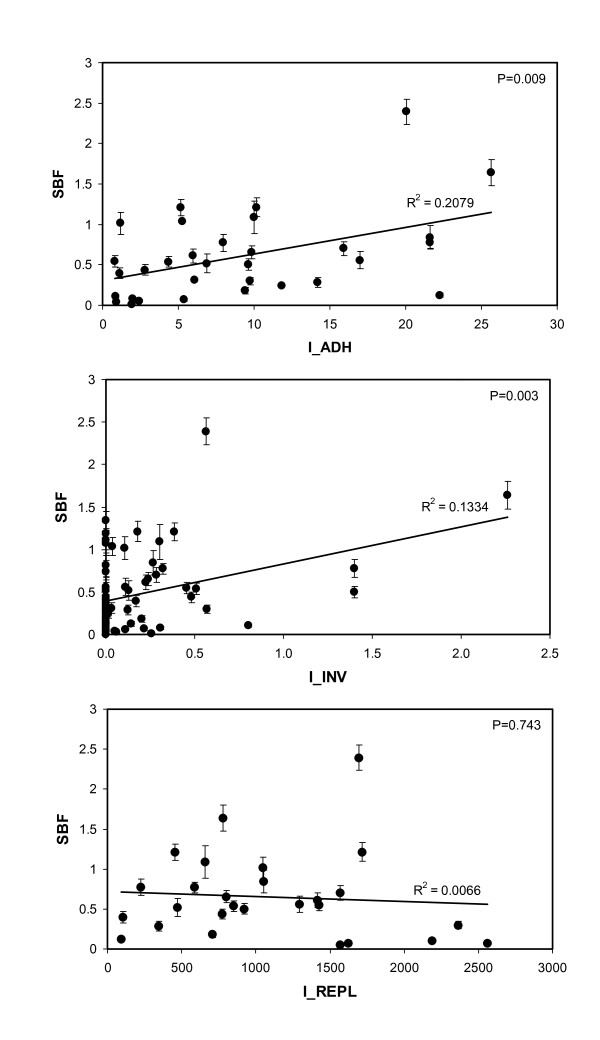
**Correlations between biofilm formation and the adhesion, invasion, and intra-macrophage replication abilities of both AIEC and non-AIEC strains**. Adhesion and invasion indices correlated positively with biofilm formation capacity, whereas intra-macrophage survival and replication did not. Adhesion index was calculated as: I_ADH = attached bacterial cells/intestinal cell; invasion index as: I_INV(%) = (intracellular bacteria/4×10^6 ^bacteria inoculated) × 100; and replication index as: I_REPL = (cfu ml^-1 ^at 24 h/cfu ml^-1 ^at 1 h)× 100.

Interestingly, higher adhesion indices from both AIEC and non-AIEC strains correlated with higher SBF indices (P = 0.009). Moreover, the correlation was even stronger between the invasion and biofilm formation capacities of AIEC strains (P = 0.003). No correlation was observed with the ability of AIEC strains to survive and replicate within macrophages (Figure 2).

### Nonmotile strains were unable to form biofilms and, amongst motile strains, those with H1 flagellar type showed the highest biofilm formation indices

An additional factor that was associated with biofilm formation was the motility of the strains. Regardless of adhesion and invasion abilities, motile strains showed higher SBF indices than nonmotile strains (SBF_MOTILE_= 0.61 ± 0.48, SBF_NONMOTILE _= 0.14 ± 0.13; P < 0.001). All strains producing moderate-strong biofilms were motile, whereas strains classified as weak biofilm producers were heterogeneous in their motility capacities. In concordance, the isogenic mutant LF82-Δ*fliC *which is nonmotile, non-flagellated and express only few type 1 pili, did not display the ability to form biofilms (SBF = 0,393 ± 0,084) in contrast to LF82 wild type (SBF = 1.641 ± 0.326).

**Figure 3:**
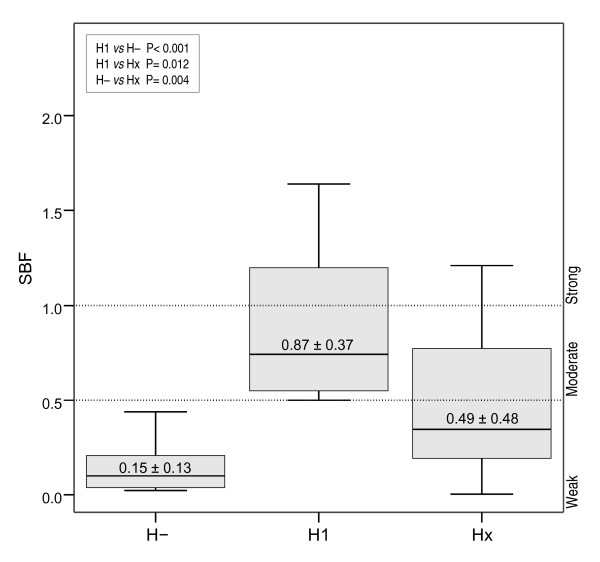
**Mean SBF index of motile and nonmotile strains irrespectively of their AIEC phenotype**. SBF indices were higher in motile strains, especially H1 serotypes, than nonmotile strains. H-: nonmotile strains; H1: motile and H1 flagellar type; Hx: motile and any flagellar type except for H1.

Moreover, SBF indices were specifically higher for the H1 serotype as shown in Figure 3. All H1 serotypes were moderate-strong biofilm producers. In contrast, only 12 out of 33 (36.4%) of strains with other H types were classified within this category (Table 3).

**Table 3:** Frequency of strains according to their motility capacity and flagellar antigen type within biofilm producers and non-producers.

	Biofilm formation category				
	**Moderate-Strong** **(N = 26)**		**Weak** **(N = 39)**		
		
**Motility/H type**	**N**	**(%)**	**N**	**(%)**	**P**

H- (N = 18)	0	(0)	18	(46.2)	< 0.001^*a*^, 0.003^*b*^

H1 (N = 14)	14	(53.8)	0	(0)	< 0.001^*a*^, < 0.001^*c*^

Hx (N = 33)	12	(46.2)	21	(53.8)	< 0.001^*c*^, 0.003^*b*^

Abbreviators: H-: nonmotile strains; H1: motile and H1 flagellar type; Hx: motile and any flagellar type except H1.^*a*^significance between H- and H1; ^*b*^significance between H- and Hx; ^*c*^significance between H1 and Hx.

To determine whether motility and AIEC-like phenotype were intrinsically related factors, the frequency of motile and nonmotile strains within AIEC and non-AIEC strains was calculated. Although the majority of AIEC strains were motile (81.5%), no significant differences were observed in comparison to non-AIEC strains (65.8%). Moreover, no interaction among these factors was detected by applying a factorial ANOVA. Therefore, motility and adherence/invasion capacity were independent factors associated with biofilm formation.

### Serogroups associated with higher biofilm producing abilities

**Figure 4:**
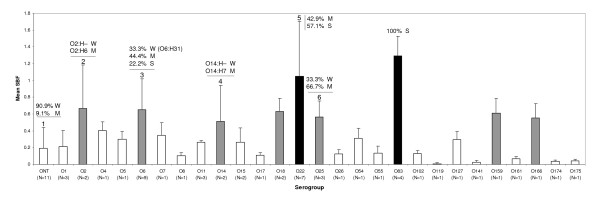
**Mean SBF index of the strains classified by their serogroup**. White bars: Serogroups with mean SBF that falls into 'weak' biofilm formation category. Grey bars: Serogroups with mean SBF that falls into 'moderate' biofilm formation category. Black bars: Serogroups with mean SBF that falls into 'strong' biofilm formation category. The serotype of those *E. coli *strains that showed different biofilm formation category than the mean SBF for the serogroup is specified: 1: Only AIEC17 (ONT:HNT) strain was classified as 'moderate' biofilm producer (M). 2: Nonmotile ECG-041 (O2:H-) strain was classified as 'weak' biofilm producer (W). 3: Three strains with O6:H31 serotype were classified as 'weak' biofilm producers, whereas strains with O6:H1, O6:H5 and O6:HNT serotypes were 'moderate' or 'strong' biofilm producers. 4: Nonmotile ECG-054 (O14:H-) was 'weak' biofilm producer (W). 5: Three strains were 'moderate' (O22:H1) and 4 strains 'strong' (O22:H1, O22:H7, and O22:H18) biofilm producers. 6: AIEC08 (O25:H4) was classified as 'weak' biofilm producer.

As shown in Figure 4, O83, followed by O22, showed the highest mean SBF indices. Regardless the AIEC phenotype and origin of the strains (intestinal or extraintestinal and non-IBD or CD associated), all the strains of O22 and O83 serogroup were found to be moderate-strong biofilm producers.

Other serogroups with mean SBF that fell into the 'moderate' category were: O2, O6, O14, O18, O25, O159, and O166. However, some strains that were unable to form biofilms were detected amongst these serogroups. For some serogroups such as O2 and O14 those strains classified as weak biofilm producers were particularly those nonmotile O2/O14 strains. In turn, strains with weak to strong biofilm formation abilities amongst the O6 strains belonged to a certain serotype (O6:H31) and this serotype was not present amongst the categories 'moderate' or 'strong' biofilm producers. Nevertheless, very few strains have been analyzed for some of these serogroups (O2, O14, O18, O25, O159, and O166) due to the nature of the strains isolated from the intestinal mucosa, thus no robust conclusions can be extracted for them.

### Distribution of virulence-associated genes and phylogroups within biofilm producers

Of the 65 *E. coli *strains used in this study, 45 (69.2%) harboured more than two virulence-associated genes in addition to *fimH*; thus, these strains are considered an extraintestinal pathogenic *E. coli *according to the definition of Johnson *et al *[21]. Virulence-associated gene distribution was similar between biofilm producers (moderate-strong) and non-biofilm producers (weak), with the exception of adherence factor *sfa/focDE *(S or F1C fimbriae) and the invasion-associated gene *ibeA *(Table 4), which were more prevalent in biofilm-forming strains (P = 0.003 and P = 0.017, respectively).

**Table 4:** Comparison of virulence gene prevalence and phylogroup between weak and moderate-strong biofilm producers.

			Biofilm formation category				
	**Total** **(N = 65)**		**Moderate-Strong** **(N = 26)**		**Weak** **(N = 39)**		**P**
		
**Virulence gene**	**N**	**(%)**	**N**	**(%)**	**N**	**(%)**	

Adhesin-encoding genes							

*papC*	32	(49.2)	11	(42.3)	21	(53.8)	0.255

*sfa/focDE*	13	(20.0)	10	(38.5)	3	(7.7)	0.003

*afa/draBC*	8	(12.3)	2	(7.7)	6	(15.4)	0.301

*fimH*	62	(95.4)	26	(100)	36	(92.3)	0.209

*fimAv*_*MT*78_	14	(21.5)	6	(23.1)	8	(20.5)	0.520

Protectin/invasion-encoding genes							

*ibeA*	9	(13.8)	7	(26.9)	2	(5.1)	0.017

K1 *neuC*	9	(13.8)	3	(11.5)	6	(15.4)	0.478

Siderophore-related genes							

*iucD*	37	(56.9)	13	(50.0)	24	(61.5)	0.253

Toxin-encoding genes							

*hlyA*	15	(23.1)	9	(34.6)	6	(15.4)	0.067

*cnf1*	15	(23.1)	9	(34.6)	6	(15.4)	0.067

*cdtB*	5	(7.7)	3	(11.5)	2	(5.1)	0.312

Phylogroup							

A	9	(13.8)	1	(3.8)	8	(21.1)	0.052

B1	8	(12.3)	3	(11.5)	5	(13.2)	0.583

B2	34	(52.3)	21	(80.8)	13	(34.2)	< 0.001

D	13	(20.0)	1	(3.8)	12	(31.6)	0.006

Although the *E. coli *collection studied was mainly composed of B2 (52.3%) and D (20%) phylotypes, significant differences were observed between the two categories of biofilm producers. As shown in Table 4, the B2 phylogroup was more frequent in moderate-strong biofilm forming strains (80.8% vs. 34.2%; P < 0.001), whereas A and D phylogroups were more frequent within weak biofilm producers.

## Discussion

In this work, we describe the biofilm formation capacity of a recently described pathovar, adherent-invasive *E. coli *(AIEC), which is associated with Crohn's disease. The main result was that AIEC strains have stronger biofilm formation abilities than other *E. coli *strains isolated from the intestinal mucosa (non-AIEC). The latter share genotypic and phenotypic traits with AIEC [15] but lack the properties described for the pathovar: (i) adhesion to and invasion of intestinal epithelial cells *in vitro*, (ii) survival and replication capacity within macrophages without causing host-cell death, and (iii) induction of TNF-α release [11]. We also analyzed the relationship between biofilm formation, AIEC phenotype, serotype, and phylogroup, and the presence of virulence-associated genes.

As observed by other authors [22,23], motility was a crucial factor for biofilm formation because none of the nonmotile strains were able to form biofilms (Table 3). This observation was further supported by the experiments performed with the isogenic mutant LF82-Δ*fliC*. Moreover, all 14 strains with H1 flagellar antigen were moderate-strong biofilm producers, in contrast to 46.2% of motile non-H1 types. Therefore, H1 flagellar antigen conferred, either directly or indirectly, an advantageous trait to form biofilms. Although motility was a necessary requirement for biofilm formation, it was not sufficient; 21 out of 47 motile strains were weak biofilm producers, indicating that additional factors are needed. In addition, strains with O2, O6, O14, O18, O22, O25, O83, O159 and O166 serogroups were found amongst the biofilm producers, in accordance with previous studies [24,25]. Interestingly, the highest mean SBFs index was achieved by four strains that belonged to the O83 serogroup, in particular the O83:H1 serotype, being all the strains classified as strong biofilm producers. This group included two AIEC strains (AIEC reference strain LF82 [11], and the sepsis-associated strain PP16) and two non-AIEC strains (ECG-009 (isolated from two different CD patients) and ECG-043 (isolated from one non-IBD control) [15].

Some associations between biofilm-formation potential and some virulence-associated genes have been already described [24,26-32]. In agreement with previous studies [25], the adhesin-coding gene *sfa/foc*DE was more frequently detected amongst biofilm producers. In addition, the gene *ibeA*, required for invasion in meningitis/sepsis-associated *E. coli *(MNEC) [33,34], was more prevalent amongst strong biofilm producers. Interestingly, *ibeA*, in conjunction with *fimH *and *fimAv*_*MT*78_, are virulence factors present in AIEC strain LF82 [16,35].

Phylogenetic analyses have shown that *E. coli *strains fall into four main phylogenetic groups (A, B1, B2, and D) and that virulent ExPEC strains mainly belong to group B2 and, to a lesser extent, group D, whereas most commensal strains belong to group A [33,36]. Although B2 was the most abundant phylotype within the *E. coli *collection, B2 phylotypes were significantly more prevalent amongst moderate-strong biofilm producers than weak biofilm producers (P < 0.001), which were enriched in A and D phylotypes (P = 0.052 and P = 0.006 respectively). Of note, B2+D phylotypes are also more prevalent amongst *E. coli *strains from patients with CD or ulcerative colitis than in non-IBD controls [37].

The positive correlation between the levels of adhesion and invasion and the higher SBF indices lead to postulate that the machinery implicated to achieve the "AIEC phenotype" could share some factors necessary for biofilm formation, such as type 1 pili and flagella. Another possibility is that both processes could be related to a coordinated expression, for instance, by the EnvZ/OmpR regulatory system. Rohlion *et al *[38] recently proposed a model in which OmpC, a porin regulated by EnvZ/OmpR, has been implicated in the adherence-invasiveness of AIEC, and this system is also known to play an important role in biofilm formation [39]. The biofilm formation could also be dependent on the cyclic di-GMP concentration which was recently reported to regulate the expression of type 1 pili and flagella in AIEC reference strain LF82 [40].

Biofilms in the human gut are thought to play an agonistic role with the host [18], being necessary to achieve an homeostatic situation and appropriate gut physiology. Nevertheless, previous studies have highlighted the increased biofilm formation in patients with CD with respect to control subjects [41]. Moreover, the composition of the mucosa-associated microbiota is altered with respect to that of non-IBD controls [42]. It is widely accepted that the intestinal microbiota is essential to elicit the inflammation; however, the specific role of intestinal biofilms in CD is still uncertain. Changes in the composition and abundance of mucosa-associated biofilms have been proposed either to play a role in the onset or perpetuation of CD [41,43-45] or to be a consequence of the defective immune regulation in CD patients [18,46,47]. Because we have analyzed the biofilm formation capacity of a collection of AIEC and non-AIEC strains using an *in vitro *method we can deduce that the ability of AIEC to form biofilms is irrespective of host factors. However, *in vivo *experiments would give interesting insights into the pathogenesis of AIEC in CD. Biofilm formation of AIEC in human gut, if confirmed, would confer to the pathovar an advantage for colonization of the intestine. Consequently, given the pathogenic behavior of AIEC, a more stable colonization would increase their probability of invading the intestinal epithelium and further trigger mucosal inflammation and, possibly, granuloma formation. In this sense, and speculatively, biofilm formation could contribute to AIEC pathogenesis.

## Conclusion

A novel phenotypic trait of AIEC pathovar was described in this work. Biofilm production ability of AIEC strains could be an additional trait involved in their pathogenesis. Further investigations to detect AIEC specific genetic determinants involved in biofilm formation and to analyze the genetic regulatory processes are essential to fully understand AIEC pathogenesis and elucidate a possible role of AIEC in CD.

## Methods

### Bacterial strains

Amongst the collection of 65 *E. coli *strains, sixty-one (93.8%) were isolated from human intestinal mucosa in previous studies [15,48]. In particular, 35 strains (16 of them were AIEC) came from CD patients, one (which belonged to AIEC pathovar) came from a patient suffering from ulcerative colitis, and 25 (of which 6 were AIEC) came from non-IBD controls. Also included were four additional AIEC strains that came from patients with extraintestinal infection (two with sepsis and two with urinary tract infection [49,50]). AIEC reference strain LF82 and the isogenic mutant LF82-Δ*fliC *were used as controls. Relevant characteristics of the strains that were known prior to this study are compiled in Table 1.

All procedures were approved by the ethics committee of clinical investigation of the Hospital Josep Trueta of Girona in compliance with the Helsinki declaration.

### Biofilm formation assay

Biofilm formation assays were performed using a previously described method [26] with some modifications [25]. Strains were grown overnight in Luria-Bertani broth with 5 g l^-1 ^of glucose (Sigma-Aldrich, St. Louis, USA) at 35.5°C, then 1/100 dilutions were made in M63 minimal medium (US Biological, Swampscott, USA) supplemented with 8 g l^-1 ^(0.8%) glucose. Then, 130-*μ*l aliquots were placed in wells of non-cell-treated polystyrene microtiter plates (Greiner Bio-one, Stuttgart, Germany) and incubated overnight at 30°C without shaking. Afterwards, growth optical densities (OD) were read at 630 nm; then the wells were washed once, adhered bacteria were stained with 1% crystal violet solubilised in ethanol, and ODs read at 570 nm. Biofilm measurements were calculated using the formula *SBF = (AB-CW)/G*, in which *SBF *is the specific biofilm formation, *AB *is the OD_570 nm _of the attached and stained bacteria, *CW *is the OD_570 nm _of the stained control wells containing only bacteria-free medium (to eliminate unspecific or abiotic OD values), and *G *is the OD_630 nm _of cell growth in broth [51,52]. For each assay, 16 wells per strain were analyzed, and the assays were performed in triplicate, which resulted in a total of 48 wells per each tested strain and control. The degree of biofilm production was classified in three categories: weak (SBF ≤ 0.5), moderate (0.5 > SBF ≤ 1), and strong (SBF > 1).

### Adhesion and invasion assays in epithelial cells Intestine-407

The epithelial cell line Intestine-407 was used for adhesion and invasion assays (ATCC accession number CCL-6™). Cell culture was performed as described previously [48]. To quantify adhesion and invasion properties, a gentamicin protection assay were performed as previously described [48]. Briefly, 24-well plates containing 4×10^5 ^cells/well incubated for 20 hours were infected at a multiplicity of infection of 10. Duplicated plates, for adhesion and invasion assays were incubated for 3 hours at 37°C. For bacterial adhesion assays, cell monolayers were washed 5 times with PBS and lysed with 1% Triton X-100. Adhered bacteria were quantified by plating them in nutrient agar. Plating was performed in a maximum period of 30 minutes to avoid bacterial lysis by Triton X-100. Adherence ability (I_ADH) was determined as the mean number of bacteria per cell. For bacterial invasion assays, monolayers were washed twice with PBS after 3 hours of infection, and fresh cell culture medium containing 100 μg ml^-1 ^of gentamicin was added for 1 hour to kill extracellular bacteria. After cell lysis with 1% Triton X-100, the number of intracellular bacteria was also determined by plating. All assays were performed in triplicate. The invasive ability was expressed as the percentage of intracellular *E. coli *compared with the initial inoculum, taken as 100%: I_INV (%) = (intracellular bacteria/4×10^6 ^bacteria inoculated) × 100.

### Survival and replication in macrophages J774

The macrophage-like J774A.1 cell line (ATCC accession number TIB-67™) was used as a model for *E. coli *survival and replication assays. Cell culture was performed as described previously [53]. *E. coli *isolates with known adherence and invasion properties were then checked for their capability to survive and replicate inside macrophages as previously described [11]. Macrophages were seeded at 2×10^5 ^cells per well in two 24-well plates and incubated for 20 hours. Once overnight medium was removed and fresh medium was added, bacteria were seeded at a multiplicity of infection of 10. Centrifugation at 900 rpm for 10 minutes, plus an additional incubation at 37°C for 10 minutes, was performed to assist the internalization of bacteria within macrophages. Non-phagocytosed bacteria were killed with gentamicin (20 μg ml^-1^), and intracellular bacteria were quantified as for invasion assays after 1 and 24 hours of infection. All assays were performed in triplicate. Results were expressed as the mean percentage of the number of bacteria recovered after 1 and 24 h post-infection compared with the initial inoculum, taken as 100%: I_REPL (%) = (cfu ml^-1 ^at 24 h/cfu ml^-1 ^at 1 h)× 100. Those strains with I_INV > 0.1 and I_REPL > 100% were classified as AIEC in this study.

### Serotyping

Determination of O and H antigens was carried out using the method previously described by Guinée *et al*. [54]. Strains which failed to achieve motility on semisolid medium were considered nonmotile and designated H-.

### Phylotyping and virulence genotyping by PCR

Determination of the major *E. coli *phylogenetic group (A, B1, B2, and D) was performed as previously described by Clermont *et al *[36].

Virulence gene carriage was analyzed as described elsewhere [25,55] using primers specific for 11 genes that encode extraintestinal virulence factors characteristic of ExPEC. These included six adhesins (pyelonephritis-associated pili (*papC*), S and F1C fimbriae (*sfa/focDE*), afimbrial Dr-binding adhesins (*afa/draBC*), type 1 fimbriae (*fimH*), and type 1 variant of avian pathogenic *E. coli *strain MT78 (*fimAv*_MT78_)); three toxins (*hlyA*, *cnf1*, and *cdtB*); and one aerobactin gene (*iucD*). They also included two protectin/invasion-encoding genes that corresponded to K1 *kps *variant (*neuC*) and brain microvascular endothelial cell invasion gene (*ibeA*). Specific genes for diarrhoeagenic *E. coli *pathovars were also screened (*stx1*, *stx2*, *eae*, *bfpA*, *ipaH*, *pCDV432*, *eltA*, and *est*).

### Statistical analyses

Quantitative parameters, such as SBF, adhesion, and invasion indices were compared by one-way ANOVA. In cases for which the interaction between several factors was of interest, a factorial ANOVA was applied. Correlation between quantitative variables was assessed by Pearson correlation coefficient. Fisher's exact test (small contingency tables) or Pearson's X^2 ^tests (frequencies higher than five within cells) were used to measure the significance of frequency values.

## Authors' contributions

MMM performed the adhesion and invasion assays, intra-macrophage survival and replication assays, statistical analyses, and drafted the manuscript. PN and CP performed the biofilm formation assays. PN also participated in drafting the manuscript. JB, JEB and MB carried out the serotyping and virulence genotyping. XA contributed by giving a medical point of view to the discussion of results. JB, FS, ADM, and JGG were involved in the design and coordination of the study, participated in the revision of the manuscript, and gave final approval of the version to be published. All authors read and approved the final version.

## References

[B1] Economou M Pappas G New Global Map of Crohn's Disease: Genetic, Environmental, and Socioeconomic Correlations Inflamm Bowel Dis 2008 14 5 709 720 10.1002/ibd.20352 18095316

[B2] Baumgart DC Carding SR Inflammatory bowel disease: cause and immunobiology Lancet 2007 369 9573 1627 1640 10.1016/S0140-6736(07)60750-8 17499605

[B3] Xavier RJ Podolsky DK Unravelling the pathogenesis of inflammatory bowel disease Nature 2007 448 7152 427 434 10.1038/nature06005 17653185

[B4] Halfvarson J Bodin L Tysk C Lindberg E Järnerot G Inflammatory bowel disease in a Swedish twin cohort: a long-term follow-up of concordance and clinical characteristics Gastroenterology 2003 124 7 1767 1773 10.1016/S0016-5085(03)00385-8 12806610

[B5] De Hertogh G Aerssens J Geboes K Geboes K Evidence for the involvement of infectious agents in the pathogenesis of Crohn's disease World J Gastroenterol 2008 14 6 845 852 10.3748/wjg.14.845 PMC2687051 18240341

[B6] Hanauer S Inflammatory Bowel Disease: Epidemiology, Pathogenesis, and Therapeutic Opportunities Inflamm Bowel Dis 2006 12 S3 S9 10.1097/01.MIB.0000195385.19268.68 16378007

[B7] Rutgeerts P Goboes K Peeters M Hiele M Penninckx F Aerts R Kerremans R Vantrappen G Effect of faecal stream diversion on recurrence of Crohn's disease in the neoterminal ileum Lancet 1991 338 8770 771 774 10.1016/0140-6736(91)90663-A 1681159

[B8] Rutgeerts P Hiele M Geboes K Peeters M Penninckx F Aerts R Kerremans R Controlled trial of metronidazole treatment for prevention of crohn's recurrence after ileal resection Gastroenterology 1995 108 6 1617 1621 10.1016/0016-5085(95)90121-3 7768364

[B9] Sartor RB Microbial Influences in Inflammatory Bowel Diseases Gastroenterology 2008 134 2 577 594 10.1053/j.gastro.2007.11.059 18242222

[B10] Sellon RK Tonkonogy S Schultz M Dieleman LA Grenther W Balish E Rennick DM Sartor RB Resident Enteric Bacteria Are Necessary for Development of Spontaneous Colitis and Immune System Activation in Interleukin-10-Deficient Mice Infect Immun 1998 66 11 5224 5231 10.1128/iai.66.11.5224-5231.1998 PMC108652 9784526

[B11] Darfeuille-Michaud A Boudeau J Bulois P Neut C Glasser A-L Barnich N Bringer M-A Swidsinski A Beaugerie L Colombel J-F High prevalence of Adherent-Invasive *Escherichia coli *Associated With Ileal Mucosa in Crohn's Disease Gastroenterology 2004 127 2 412 421 10.1053/j.gastro.2004.04.061 15300573

[B12] Martin HM Campbell BJ Hart CA Mpofu C Nayar M Singh R Englyst H Williams HF Rhodes JM Enhanced *Escherichia coli *adherence and invasion in Crohn's disease and colon cancer Gastroenterology 2004 127 1 80 93 10.1053/j.gastro.2004.03.054 15236175

[B13] Baumgart M Dogan B Rishniw M Weitzman G Bosworth B Yantiss R Orsi RH Wiedmann M McDonough P Kim SG Berg D Schukken Y Scherl E Simpson KW Culture independent analysis of ileal mucosa reveals a selective increase in invasive *Escherichia coli *of novel phylogeny relative to depletion of Clostridiales in Crohn's disease involving the ileum ISME J 2007 1 5 403 418 10.1038/ismej.2007.52 18043660

[B14] Sasaki M Sitaraman SV Babbin BA Gerner-Smidt P Ribot EM Garrett N Alpern JA Akyildiz A Theiss AL Nusrat A Klapproth J-MA Invasive *Escherichia coli *are a feature of Crohn's disease Lab Invest 2007 87 10 1042 1054 10.1038/labinvest.3700661 17660846

[B15] Martinez-Medina M Aldeguer X Lopez-Siles M González-Huix F López-Oliu C Dahbi G Blanco JE Blanco J Garcia-Gil LJ Darfeuille-Michaud A Molecular diversity of *Escherichia coli *in the human gut: new ecological evidence supporting the role of adherent-invasive *E. coli *(AIEC) in Crohn's disease Inflamm Bowel Dis 2009 15 6 872 882 10.1002/ibd.20860 19235912

[B16] Simpson KW Dogan B Rishniw M Goldstein RE Klaessig S McDonough PL German AJ Yates RM Russell DG Johnson SE Berg DE Harel J Bruant G McDonough SP Schukken YH Adherent and Invasive *Escherichia coli *Is Associated with Granulomatous Colitis in Boxer Dogs Infect Immun 2006 74 8 4778 4792 10.1128/IAI.00067-06 PMC1539603 16861666

[B17] Hall-Stoodley L Stoodley P Biofilm formation and dispersal and the transmission of human pathogens Trends Microbiol 2005 13 1 7 10 10.1016/j.tim.2004.11.004 15639625

[B18] Everett ML Palestrant D Miller SE Bollinger RR Parker W Immune exclusion and immune inclusion: a new model of host-bacterial interactions in the gut Clinical Applied Imm Rev 2004 4 5 321 332 10.1016/j.cair.2004.03.001

[B19] Anderson GG Palermo JJ Schilling JD Roth R Heuser J Hultgren SJ Intracellular bacterial biofilm-like pods in urinary tract infections Science 2003 301 5629 105 107 10.1126/science.1084550 12843396

[B20] Kaper JB Nataro JP Mobley HLT Pathogenic *Escherichia coli* Nat Rev Microbiol 2004 2 2 123 140 10.1038/nrmicro818 15040260

[B21] Johnson JR Murray AC Gajewski A Sullivan M Snippes P Kuskowski MA Smith KE Isolation and Molecular Characterization of Nalidixic Acid-Resistant Extraintestinal Pathogenic *Escherichia coli *from Retail Chicken Products Antimicrob Agents Chemother 2003 47 7 2161 2168 10.1128/AAC.47.7.2161-2168.2003 PMC161843 12821463

[B22] Pratt LA Kolter R Genetic analysis of *Escherichia coli *biofilm formation: roles of flagella, motility, chemotaxis and type I pili Mol Microbiol 1998 30 2 285 293 10.1046/j.1365-2958.1998.01061.x 9791174

[B23] Van Houdt R Michiels CW Role of bacterial cell surface structures in *Escherichia coli *biofilm formation Res Microbiol 2005 156 5-6 626 633 10.1016/j.resmic.2005.02.005 15950122

[B24] Kanamaru S Kurazono H Terai A Monden K Kumon H Mizunoe Y Ogawa O Yamamoto S Increased biofilm formation in *Escherichia coli *isolated from acute prostatitis Int J Antimicrob Agents 2006 28 Supplement 1 21 25 10.1016/j.ijantimicag.2006.05.006 16828264

[B25] Naves P del Prado G Huelves L Gracia M Ruiz V Blanco J Dahbi G Blanco M del Carmen Ponte M Soriano F Correlation between virulence factors and in vitro biofilm formation by *Escherichia coli *strains Microb Pathog 2008 45 2 86 91 10.1016/j.micpath.2008.03.003 18486439

[B26] Danese P Pratt L Dove S Kolter R The outer membrane protein, Antigen 43, mediates cell-to-cell interactions within *Escherichia coli *biofilms Mol Microbiol 2000 37 2 424 432 10.1046/j.1365-2958.2000.02008.x 10931336

[B27] Ong C-LY Ulett GC Mabbett AN Beatson SA Webb RI Monaghan W Nimmo GR Looke DF McEwan AG Schembri MA Identification of Type 3 Fimbriae in Uropathogenic *Escherichia coli *Reveals a Role in Biofilm Formation J Bacteriol 2008 190 3 1054 1063 10.1128/JB.01523-07 PMC2223576 18055599

[B28] Schembri MA Dalsgaard D Klemm P Capsule Shields the Function of Short Bacterial Adhesins J Bacteriol 2004 186 5 1249 1257 10.1128/JB.186.5.1249-1257.2004 PMC344426 14973035

[B29] Soto SM Smithson A Martinez JA Horcajada JP Mensa J Vila J Biofilm Formation in Uropathogenic *Escherichia coli *Strains: Relationship With Prostatitis, Urovirulence Factors and Antimicrobial Resistance J Urol 2007 177 1 365 368 10.1016/j.juro.2006.08.081 17162092

[B30] Ulett GC Mabbett AN Fung KC Webb RI Schembri MA The role of F9 fimbriae of uropathogenic *Escherichia coli *in biofilm formation Microbiology 2007 153 7 2321 2331 10.1099/mic.0.2006/004648-0 17600076

[B31] Ulett GC Valle J Beloin C Sherlock O Ghigo J-M Schembri MA Functional Analysis of Antigen 43 in Uropathogenic *Escherichia coli *Reveals a Role in Long-Term Persistence in the Urinary Tract Infect Immun 2007 75 7 3233 3244 10.1128/IAI.01952-06 PMC1932929 17420234

[B32] Vianney A Jubelin G Renault S Dorel C Lejeune P Lazzaroni JC *Escherichia coli tol *and *rcs *genes participate in the complex network affecting curli synthesis Microbiology 2005 151 7 2487 2497 10.1099/mic.0.27913-0 16000739

[B33] Bidet P Mahjoub-Messai F Blanco J Blanco J Dehem M Aujard Y Bingen E Bonacorsi S Combined multilocus sequence typing and O serogrouping distinguishes *Escherichia coli *subtypes associated with infant urosepsis and/or meningitis J Infect Dis 2007 196 2 297 303 10.1086/518897 17570118

[B34] Xie Y Kim KJ Kim KS Current concepts on *Escherichia coli *K1 translocation of the blood-brain barrier FEMS Immunol Med Microbiol 2004 42 3 271 279 10.1016/j.femsim.2004.09.001 15477040

[B35] Boudeau J Barnich N Darfeuille-Michaud A Type 1 pili-mediated adherence of *Escherichia coli *strain LF82 isolated from Crohn's disease is involved in bacterial invasion of intestinal epithelial cells Mol Microbiol 2001 39 5 1272 1284 10.1111/j.1365-2958.2001.02315.x 11251843

[B36] Clermont O Bonacorsi S Bingen E Rapid and Simple Determination of the *Escherichia coli *Phylogenetic Group Appl Environ Microbiol 2000 66 10 4555 4558 10.1128/AEM.66.10.4555-4558.2000 PMC92342 11010916

[B37] Kotlowski R Bernstein CN Sepehri S Krause DO High prevalence of *Escherichia coli *belonging to the B2+D phylogenetic group in inflammatory bowel disease Gut 2007 56 5 669 675 10.1136/gut.2006.099796 PMC1942160 17028128

[B38] Rolhion N Carvalho FA Darfeuille-Michaud A OmpC and the sigma(E) regulatory pathway are involved in adhesion and invasion of the Crohn's disease-associated *Escherichia coli *strain LF82 Mol Microbiol 2007 63 6 1684 1700 10.1111/j.1365-2958.2007.05638.x 17367388

[B39] Pruss BM Besemann C Denton A Wolfe AJ A Complex Transcription Network Controls the Early Stages of Biofilm Development by *Escherichia coli* J Bacteriol 2006 188 11 3731 3739 10.1128/JB.01780-05 PMC1482888 16707665

[B40] Claret L Miquel S Vieille N Ryjenkov DA Gomelsky M Darfeuille-Michaud A The flagellar sigma factor FliA regulates adhesion and invasion of Crohn disease-associated *Escherichia coli *via a cyclic dimeric GMP-dependent pathway J Biol Chem 2007 282 46 33275 33283 10.1074/jbc.M702800200 17827157

[B41] Swidsinski A Ladhoff A Pernthaler A Swidsinski S Loening-Baucke V Ortner M Weber J Hoffmann U Schreiber S Dietel M Lochs H Mucosal flora in inflammatory bowel disease Gastroenterology 2002 122 1 44 54 10.1053/gast.2002.30294 11781279

[B42] Martinez-Medina M Aldeguer X Gonzalez-Huix F Acero D Garcia-Gil LJ Abnormal microbiota composition in the ileocolonic mucosa of Crohn's disease patients as revealed by polymerase chain reaction-denaturing gradient gel electrophoresis Inflamm Bowel Dis 2006 12 12 1136 1145 10.1097/01.mib.0000235828.09305.0c 17119388

[B43] Dicksved J Halfvarson J Rosenquist M Jarnerot G Tysk C Apajalahti J Engstrand L Jansson JK Molecular analysis of the gut microbiota of identical twins with Crohn's disease ISME J 2008 2 7 716 727 10.1038/ismej.2008.37 18401439

[B44] Kleessen B Kroesen A Buhr H Blaut M Mucosal and invading bacteria in patients with inflammatory bowel disease compared with controls Scand J Gastroenterol 2002 37 9 1034 1041 10.1080/003655202320378220 12374228

[B45] Schultsz C Berg FM van den ten Kate FW Tytgat GNJ Dankert J The intestinal mucus layer from patients with inflammatory bowel disease harbors high numbers of bacteria compared with controls Gastroenterology 1999 117 5 1089 1097 10.1016/S0016-5085(99)70393-8 10535871

[B46] Lupp C Robertson ML Wickham ME Sekirov I Champion OL Gaynor EC Finlay BB Host-mediated inflammation disrupts the intestinal microbiota and promotes the overgrowth of *Enterobacteriaceae* Cell Host Microbe 2007 2 2 119 129 10.1016/j.chom.2007.06.010 18005726

[B47] Wehkamp J Stange EF Is there a role for defensins in IBD? Inflamm Bow Dis 2008 14 S2 S85 S87 10.1002/ibd.20698 18816709

[B48] Boudeau J Glasser A-L Masseret E Joly B Darfeuille-Michaud A Invasive ability of an *Escherichia coli *strain isolated from the ileal mucosa of a patient with Crohn's disease Infect Immun 1999 67 9 4499 4509 10.1128/iai.67.9.4499-4509.1999 PMC96770 10456892

[B49] Blanco M Blanco JE Alonso MP Mora A Balsalobre C Munoa F Juárez A Blanco J Detection of pap, sfa and afa adhesin-encoding operons in uropathogenic *Escherichia coli *strains: Relationship with expression of adhesins and production of toxins Res Microbiol 1997 148 9 745 755 10.1016/S0923-2508(97)82450-3 9765858

[B50] Nicolas-Chanoine M-H Blanco J Leflon-Guibout V Demarty R Alonso MP Canica MM Park Y-J Lavigne J-P Pitout J Johnson JR Intercontinental emergence of *Escherichia coli *clone O25:H4-ST131 producing CTX-M-15 J Antimicrob Chemother 2008 61 2 273 281 10.1093/jac/dkm464 18077311

[B51] Naves P Prado Gd Huelves L Gracia M Ruiz V Blanco J Rodríguez-Cerrato V Ponte MC Soriano F Measurement of biofilm formation by clinical isolates of *Escherichia coli *is method-dependent J Appl Microbiol 2008 105 2 585 590 10.1111/j.1365-2672.2008.03791.x 18363684

[B52] Niu C Gilbert ES Colorimetric Method for Identifying Plant Essential Oil Components That Affect Biofilm Formation and Structure Appl Environ Microbiol 2004 70 12 6951 6956 10.1128/AEM.70.12.6951-6956.2004 PMC535164 15574886

[B53] Glasser A-L Boudeau J Barnich N Perruchot M-H Colombel J-F Darfeuille-Michaud A Adherent Invasive *Escherichia coli *Strains from Patients with Crohn's Disease Survive and Replicate within Macrophages without Inducing Host Cell Death Infect Immun 2001 69 9 5529 5537 10.1128/IAI.69.9.5529-5537.2001 PMC98666 11500426

[B54] Guinée PA Agterberg CM Jansen WH *Escherichia coli *O antigen typing by means of a mechanized microtechnique Appl Microbiol 1972 24 1 127 131 10.1128/am.24.1.127-131.1972 PMC380558 4560465

[B55] Blanco M Blanco J Dahbi G Alonso M Mora A Coira M Madrid C Juárez A Bernárdez M González E Blanco J Identification of two new intimin types in atypical enteropathogenic *Escherichia coli* Int Microbiol 2006 9 2 103 110 16835840

